# Understanding the biology and use of anti-TNF agents in JIA – interim results

**DOI:** 10.1186/1546-0096-10-S1-A48

**Published:** 2012-07-13

**Authors:** Daniel J Lovell, Steven P Spalding, Karen Onel, Beth S Gottlieb, Hermine I Brunner, Yukiko Kimura, Paula W Morris, Judyann C Olson, Anne Johnson, Edward H Giannini

**Affiliations:** 1 Cincinnati Childrens Hospital Medical Center, Cincinnati, OH, USA; 2 Cleveland Clinic, Cleveland, OH, USA; 3 Hackensack University Medical Center, Hackensack, NJ, USA; 4 Medical College of Wisconsin, Milwaukee, WI, USA; 5 Schneider Children's Hospital, New Hyde Park, NY, USA; 6 University of Arkansas for Medical Science, Little Rock, AR, USA; 7 University of Chicago, Chicago, IL, USA

## Purpose

Current treatment for children with polyarticular forms of Juvenile Idiopathic Arthritis (Poly JIA) results in approximately 50% of the patients demonstrating clinically inactive disease (CID) (Wallace C, et al. J Rheumatol 2004;31:2290-4) while on treatment. Over 40% of the children with Poly JIA are treated with a TNF antagonist biologic often started ≤ 6 months (mos) of disease onset. Currently we are unable to accurately predict which children demonstrating CID while on an anti-TNF therapy will or will not have a flare of the JIA upon discontinuation of the anti-TNF agent. Anti-TNF therapy has known short- and medium-term toxicities in children with JIA; the long-term toxicities are unknown and the treatment is expensive. The ultimate goal of this project is to validate biomarkers that are predictive. These interim results demonstrate clinical aspects of assessing CID and stopping anti-TNF therapy.

## Methods

In 12 pediatric rheumatology centers, 120 children with Poly JIA demonstrating CID while on anti-TNF therapy will be enrolled and followed for up to 14 mos. In those subjects who demonstrate CID for the first 6 mos of the study, the anti-TNF therapy will be stopped. The primary outcome variable (POV) is disease flare within the next 8 months using a validated definition of disease flare (Brunner HI, et al. J Rheumatol 2002;29:1058-64).

## Results

Forty eight subjects were enrolled by 12/20/2010. At the time of enrollment, the mean (median) age was 11.5 yrs (12.4) and disease duration 5.9 yrs (4.4). JIA subtype was Extended Oligo in 5, Poly RF negative in 38, and Poly RF positive in 5. 20 subjects were ANA positive, 27 ANA negative and 1 unknown. There were 35 females and 14 males; 45 were Caucasian and 3 African-American; 3 were Hispanic. Thirty eight (79%) were taking etanercept, 7 (15%) adalimumab and 3 (6%) infliximab. Sixteen (33%) were taking background methotrexate, while 32 (66%) were on anti-TNF therapy monotherapy. At the baseline study visit, the mean (median) duration of anti-TNF therapy was 2.2 yrs (1.5). All 48 subjects met the criteria for CID at the base line visit. At the time of analysis, 7/48 (14%) failed to demonstrate continued CID for the first 6 months of the study. Eighteen subjects demonstrated CID for the first 6 mos of the study and stopped anti-TNF therapy at the month 6 study visit. One subject discontinued the study due to logistical reasons (relocation of the family). In the 18 subjects stopping anti-TNF therapy, 6 (33%) have flared at a mean (median) of 56 days (28) after stopping the anti-TNF therapy. In the 12 subjects not demonstrating flare the mean (median) time off the anti-TNF agent is 143 days (158). This study is ongoing and the other 23 subjects have not yet completed the first 6 mos of the study.

## Conclusion

In children with one of the polyarticular forms of JIA on stable anti-TNF therapy, having achieved CID at one visit, approximately 14% will not remain in CID for the next 6 months. In those who stop the anti-TNF therapy, 66% have remained off the anti-TNF therapy without demonstrating flare to date.

## Disclosure

Daniel J. Lovell: Abbott Immunology Pharmaceuticals, 5, Amgen Inc., 5, Centocor, Inc., 5, UCB, Inc., 5; Steven P. Spalding: None; Karen Onel: None; Beth S. Gottlieb: None; Hermine I. Brunner: Abbott Laboratories, 5, Amgen Inc., 5, Centocor, Inc., 5, UCB, Inc., 5; Yukiko Kimura: None; Paula W. Morris: None; Judyann C. Olson: Abbott Laboratories, 2; Anne Johnson: None; Edward H. Giannini: None.

